# Does Asthma Increase the Odds of Suicidal Thoughts, Plans, and Attempts?

**DOI:** 10.7759/cureus.53865

**Published:** 2024-02-08

**Authors:** Karan Varshney, Pavan Shet, Brandon George, Matthew Wintersteen

**Affiliations:** 1 Public Health, School of Medicine, Deakin University, Waurn Ponds, AUS; 2 Internal Medicine, School of Medicine, Deakin University, Waurn Ponds, AUS; 3 Jefferson College of Population Health, Thomas Jefferson University, Philadelphia, USA; 4 Sidney Kimmel Medical College, Thomas Jefferson University, Philadelphia, USA

**Keywords:** allergic comorbid conditions, social determinants of health (sdoh), epidemiology, suicide risk, asthma

## Abstract

Background

Asthma is a chronic inflammatory disease of the airways affecting more than 250 million people worldwide. In the past, a possible relationship between asthma and suicidality has been hypothesized. However, further research is required as this link has not been clearly established. Our objective was to use propensity score matching to answer the following research question: does having asthma increase one’s odds of developing suicidality throughout their lifetime and, if so, how large is this increase?

Methodology

We utilized data from the 2018 National Survey on Drug Use and Health. We analyzed the relationship between currently having asthma and having had suicidal thoughts, suicide plans, and suicide attempts over the past 12 months. Chi-square analyses were performed both before and after completing propensity score matching.

Results

Before matching, it was found that, compared to individuals without asthma, asthmatic individuals had 31.2% higher odds of having suicidal thoughts (p = 0.010) and 97.4% higher odds of a suicide attempt (p = 0.012). After controlling for confounders by matching, there was no longer a relationship between having asthma and suicidal thoughts (p = 0.707), suicidal plans (p = 0.523), and suicidal attempts (p = 0.260).

Conclusions

These findings highlight that while asthma may appear to be associated with suicidality, this association does not persist after controlling for confounding factors. Hence, it is recommended that more research be conducted on this topic and that possible confounders be further researched. In particular, there is a need to better understand the role of social determinants and other contributors to health outcomes.

## Introduction

Asthma is a serious, chronic inflammatory disease of the airways that impacts a high number of individuals. Asthma impacts more than 250 million people worldwide, and, in 2019, was a direct cause of approximately 455,000 deaths [1]. Across the United States, there are more than 25 million people who are diagnosed with asthma, meaning that about one in 13 people in the country currently have the disease [2]. While asthma is known to be a disease that primarily affects one’s respiratory function, its effects can also severely hamper one’s quality of life. This is demonstrated by evidence indicating that asthma may increase one’s vulnerability to mental health issues and depressive disorders. For example, one study that used data from more than 15,000 questionnaire participants showed that asthma patients are considerably more likely to deal with, and report, frequent mental distress [3]. A separate cross-sectional survey involving about 3,500 individuals indicated that those with asthma may be at a greater risk of dealing with a mental health condition in comparison to those without asthma [4]. Other large studies and reviews have also demonstrated a link between asthma and mental health disorders, although there have been inherent difficulties with determining causality; some mental health disorders that appear to be more common among asthmatics compared to the general population are anxiety, panic disorder/agoraphobia, post-traumatic stress disorder, social phobia, dysthymia, major depressive disorder, and alcohol dependence [5,6].

In addition to the literature linking asthma and mental health issues, there is some research showing a possible link between asthma and suicidality, although this link has not yet been clearly established [7,8]. A prior systematic review [8] analyzed 19 articles and found that there may be a potential link between asthma and suicidality, although there is a need for more research on the topic as several included studies did not find such an association [9-11]. A systematic review and meta-analysis published in 2019 also demonstrated that asthmatic patients had a higher risk for suicidal ideation, suicidal attempts, and suicide mortality [12].

While there is evidence available demonstrating a possible link between asthma and suicidality, it is important to emphasize that a sizeable number of additional studies on this topic have failed to find such an association [13-18]. Of these studies, the one with the largest sample size had data on a total of 683,716 participants and found equal rates of suicidality among those who have ever had asthma and those who have never had asthma [13].

Due to the inconsistencies in the literature, there is currently a need for further research to be conducted to better determine the extent to which an asthma diagnosis will increase one’s likelihood of developing suicidality at some point (or if it increases one’s likelihood at all). Based on this need for better establishing a possible link between asthma and suicidality, this project will aim to answer the following question: does having asthma increase one’s odds of developing suicidal thoughts, plans, or behaviors, and to what degree?

## Materials and methods

This cross-sectional analysis utilized observational data from the 2018 National Survey on Drug Use and Health (NSDUH) [19]. NSDUH is a survey regarding substance abuse and mental health status and was administered to people across the United States aged 12 years or older. More precisely, this survey was conducted across all 50 American states, as well as the District of Columbia, with household addresses being selected randomly. The overall purpose of this survey is to provide information regarding the patterns in substance usage, as well as in mental health and other issues relating to public health, across the United States [19]. In total, data from 67,791 individuals were obtained from citizens across the country [19].

One of the many questions asked on the national survey was whether one is living with asthma (yes/no). Questions on the topic of suicidality involved asking whether, over the past year, one has had serious thoughts of suicide, made a suicide plan, or attempted suicide (all three of these separate questions regarding suicide had yes/no responses). Only those who responded “yes” to the question regarding suicidal ideation in the survey were asked about suicide plans and suicide attempts. Due to the differences in the types of questions provided to youth and adults, only responses provided by adults (those aged 18 years or above) were included in this analysis. Only adults who provided a “yes” or a “no” response to all of the following questions were eligible for inclusion in our analyses: (1) do you currently have asthma? (2) Have you seriously considered suicide in the past 12 months? (3) Have you made a suicide plan in the past 12 months? (4) Have you attempted suicide in the past 12 months?

Two different analyses were conducted to analyze the potential relationship between asthma and suicidality. The first analysis involved conducting three different chi-square tests between having asthma, including either having had serious thoughts of suicide in the past 12 months, having had plans of suicide over the past 12 months, or having attempted suicide over the past 12 months. The first analysis did not involve controlling for any covariates.

The second analysis involved first using propensity score matching to control for possible confounding variables that may have influenced the results. Propensity score matching was chosen for this study as it has been a frequently utilized statistical technique that has been shown to be capable of reducing the impact of bias and confounding variables [20]. Numerous covariates chosen were based on demographic factors that may influence outcomes, as well as numerous other variables that were chosen based on the possibility that they may influence one’s mental health and, hence, one’s probability of developing suicidality. More specifically, the variables included in the analysis are those pertaining to age in years (18-25, 26-34, 35-49, 50+), race (White, Black, Native American, Asian, Hispanic, multi-race), sex (male, female), sexual identity (heterosexual, lesbian/gay, bisexual), and marital status (married, widowed, divorced/separated, never been married), as well as education status (currently undergoing education, not currently undergoing education), educational attainment (less than high school, high school graduate, some college, college graduate), socioeconomic status (<$20,000, $20,000-49,999, $50,000-74,999, $75,000+), substance usage (yes, no), overall health status (poor, fair, good, very good, excellent), Medicare coverage (yes, no), belief that religion is very important (strongly disagree, disagree, agree, strongly agree), if religion influences decision-making (strongly disagree, disagree, agree, strongly agree), ever incarcerated (yes, no), and having ever been in the armed forces (yes, no).

Matching occurred with a match tolerance of 0.05. The propensity score matching tolerance was chosen at the level of 0.05 to ensure minimal differences in scores between the control and treatment groups. After matching, three chi-square tests, which analyzed the same variables regarding suicide and asthma as those of the first analysis, were conducted. The data analyses were done using SAS (SAS Institute Inc., Cary, NC, USA), with propensity score matching done via PROC PSMATCH.

## Results

Table 1 shows the frequency of responses to questions regarding asthma and suicidal ideation, plans, and attempts. While 42,685 individuals responded to the question regarding suicidal ideation over the past year, only 4,528 responded to both this question and the question regarding currently having asthma. Therefore, for the first analysis which occurred without covariates, a maximum of 4,528 participants were included.

**Table 1:** Total yes/no responses for questions on asthma and suicidality before matching.

Question	Yes (%)	No (%)	Total
Do you currently have asthma?	4,035 (64.7)	2,203 (35.3)	6,238
Did you seriously consider suicide in the past year?	2,739 (6.4)	39,946 (93.6)	42,685
Did you make a suicide plan in the past year?	892 (32.7)	1,838 (67.3)	2,730
Did you attempt suicide in the past year?	420 (15.4)	2,313 (84.6)	2,733

The results of both analyses, before and after matching, are summarized in Table 2. In the analysis which was done before matching, an association between having asthma and having suicidal thoughts was found (p = 0.010) as those living with asthma had 31.2% higher odds of having suicidal thoughts compared to those without asthma (95% confidence interval (CI) = 1.069, 1.611). There was also an association (p = 0.012) between asthma and having made a suicide attempt, with those living with asthma having 97.4% higher odds of attempting suicide (95% CI = 1.147, 3.397). An association between having an asthma diagnosis and having a suicide plan was not found (odds ratio (OR) = 1.397, 95% CI = 0.998, 1.976; p = 0.057).

**Table 2:** Odds of suicidal behavior among those with asthma compared to those without asthma before matching and after matching. OR: odds ratio; CI: confidence interval

	Before matching		After matching	
	OR (95% CI)	P-value	OR (95% CI)	P-value
Suicidal thoughts	1.312 (1.069, 1.611)	0.010	1.056 (0.826, 1.350)	0.707
Suicide plan	1.397 (0.998, 1.976)	0.057	1.147 (0.753, 1.747)	0.523
Suicide attempt	1.974 (1.147, 3.397)	0.012	1.443 (0.759, 2.744)	0.260

After matching, there was no association between having an asthma diagnosis and having suicidal thoughts (OR = 1.056, 95% CI = 0.826, 1.350; p = 0.707). There was no association between ever having been diagnosed with asthma and ever having a suicide plan (OR = 1.147, 95% CI = 0.753, 1.747; p = 0.523). Lastly, there was no association between being an asthmatic and having attempted suicide (OR = 1.443; 95% CI = 0.759, 2.744; p = 0.260).

## Discussion

This study found that, without any covariates in the analysis, there was an association between living with asthma and suicidality, with a particularly strong association with suicide attempts; this initially seemed to provide some evidence for the existence of this relationship. However, this association disappeared when analysis was conducted using propensity score matching to control for covariates. Therefore, it appears that there may be factors other than an asthma diagnosis that are responsible for the association or that contribute to the association. Based on our findings, future studies must focus on the link between asthma and suicidal behavior and account for demographic factors, as well as social determinants of health, which include socioeconomic status, educational attainment, overall health status, and substance use. Prior studies have largely not accounted for such factors, which may have influenced the overall findings. Hence, our work highlights the importance of the usage of statistical methods, such as propensity score matching, in determining and considering covariates when analyzing the relationship between asthma and suicidality. Furthermore, our findings show that, when clinicians consider the risk for asthma patients developing further comorbidities, they must consider social determinants of health for patients, especially as these may potentially have an important role in increasing (or decreasing) the risk of developing mental health problems such as suicidal thoughts and other forms of psychopathology.

The overall findings of this study seem to deviate from the findings of several previous studies. This has been demonstrated in a prior review [8], which found that only three out of the 19 studies that were reviewed did not find an association between asthma and suicidality after controlling for confounders [9-11]. In such studies, comorbidities, low income, and depression were shown to be important confounding variables in the relationship between suicidal behavior and having asthma. Based on this, as well as the existence of numerous other studies that did not find an association [13-17], it is recommended that more research be conducted in the future to better understand the relationship between asthma and suicidality, with consideration of confounders or mediators of this relationship.

There are numerous limitations of this work that need to be acknowledged. First, as many individuals did not respond to the outcome variables, there was a notable amount of missing data in the analysis. It is important to recognize that a large number of respondents chose not to answer questions regarding suicidality and asthma and that there may have been social desirability bias, where individuals chose not to give truthful responses out of fear of being judged; this missing data may have influenced the overall findings of this work, which are reliant on a survey that utilizes self-reporting. Second, due to the cross-sectional nature of this survey, this study was only able to account for risks at specific points in one’s lifetime. Furthermore, this analysis did not account for the severity of each respondent’s asthma, nor the duration for which they had the disease. These factors would have likely impacted the respondent’s mental health and would have been worthwhile to account for in an analysis. However, this was not possible in this study. It should also be acknowledged that numerous other health-related confounding variables, which may have been relevant, could not be considered in our analyses.

Regardless of the limitations, our work has several notable strengths. Our usage of propensity score matching provides more nuance to the current understanding of the relationship between asthma and suicide and highlights that there are important confounding variables that may be influencing this relationship. Moreover, the sample size for this study was large enough to show statistically relevant trends. In terms of overall implications, this work indicates that a relationship between asthma and overall suicide risk may appear to exist, but might not persist after one can appropriately account for the influence of relevant covariates. More research is needed to better understand the complex relationship between asthma and suicidality. Future research focusing on this relationship may also provide valuable insights regarding asthma’s link to comorbidities in general, as the current understanding of the relationship between asthma and conditions such as reflux, hormonal disorders, and psychopathologies is poor [21].

## Conclusions

Our study has shown that, while there initially appeared to be a link between having asthma and living with suicidality, this association was no longer present after controlling for demographic factors and social determinants of health. It was shown that before propensity score matching, there was a statistical association between currently having asthma and having had recent suicidal thoughts and attempts. However, after controlling for a large number of demographic and health-related covariates, no statistical relationship persisted between asthma and suicidal thoughts, planning, or attempts.

The findings from our study differ from those of numerous past studies, which instead showed a potential association between having asthma and having some form of suicidality. Overall, these discrepancies across studies highlight the significance of using propensity score matching, as well as other statistical approaches such as penalized regression, and the Cochran-Mantel-Haenszel method, to better understand the relationship between asthma and suicidality. More research is needed to better understand the extent to which specific confounders influence this relationship. It is recommended that future research explores these factors to better understand the relationship between asthma and suicidality, as well as to better understand the social and lived realities that may be contributing to the mental health problems of asthmatics. This will be important in improving long-term overall health outcomes for those living with asthma.
